# Restricted immunological and cellular pathways are shared by murine models of chronic alcohol consumption

**DOI:** 10.1038/s41598-020-59188-9

**Published:** 2020-02-12

**Authors:** Alyx Vogle, Tongqi Qian, Shijia Zhu, Elizabeth Burnett, Holger Fey, Zhibin Zhu, Ali Keshavarzian, Maliha Shaikh, Yujin Hoshida, Miran Kim, Costica Aloman

**Affiliations:** 1 Division of Digestive Diseases and Nutrition, Section of Hepatology, Rush University, Chicago, IL USA; 2 University of Texas Southwestern Medical Center, Division of Digestive Diseases, Department of Internal Medicine, Texas, USA

**Keywords:** Alcoholic liver disease, Chronic inflammation

## Abstract

Murine models of chronic alcohol consumption are frequently used to investigate alcoholic liver injury and define new therapeutic targets. Lieber-DeCarli diet (LD) and Meadows-Cook diet (MC) are the most accepted models of chronic alcohol consumption. It is unclear how similar these models are at the cellular, immunologic, and transcriptome levels. We investigated the common and specific pathways of LD and MC models. Livers from LD and MC mice were subjected to histologic changes, hepatic leukocyte population, hepatic transcripts level related to leukocyte recruitment, and hepatic RNA-seq analysis. Cross-species comparison was performed using the alcoholic liver disease (ALD) transcriptomic public dataset. Despite LD mice have increased liver injury and steatosis by alcohol exposure, the number of CD45^+^ cells were reduced. Opposite, MC mice have an increased number of monocytes/liver by alcohol. The pattern of chemokine gradient, adhesion molecules, and cytokine transcripts is highly specific for each model, not shared with advanced human alcoholic liver disease. Moreover, hepatic RNA-seq revealed a limited and restricted number of shared genes differentially changed by alcohol exposure in these 2 models. Thus, mechanisms involved in alcohol tissue injury are model-dependent at multiple levels and raise the consideration of significant pathophysiological diversity of human alcoholic liver injury.

## Introduction

Murine models are frequently used to investigate new pathophysiological pathways of human diseases and many new treatment trials for alcoholic liver disease (ALD) are based on preclinical testing provided by murine models.

ALD in humans is the result of a complex interaction between the alcoholic effects on gut microbiota, intestinal cells, immune system, and the hepatic microenvironment^1,2^. Based on an extensive body of literature, alcohol increases the intestinal permeability and subsequent systemic translocation of bacterial products, e.g. LPS^1,3,4^. Stimulation of Toll-like receptors (TLR) by bacterial products results in immune cell activation related to hepatic alcohol metabolic process that has a deleterious effect on the liver, resulting in histological features of ALD: steatosis, innate immune infiltrate with predominance of neutrophils and monocytes, and histological signs of hepatocyte dysfunction (Mallory hyaline formation, ballooning hepatocyte)^3,5–7^. ALD includes a spectrum of histological and clinical entities: steatosis, steatohepatitis, and alcoholic hepatitis^8,9^. In time, chronic liver injury with a dysregulated innate immune response results in alcohol-induced progressive hepatic fibrogenesis, liver cirrhosis and end-stage liver disease^10^.

Based on this classical paradigm, few trials have been attempted to control an exacerbated innate immune response or to improve hepatocyte function. Besides steroids that may be beneficial for only subsets of patients with alcoholic hepatitis^11^, no other treatments have proven consistent survival benefit^12^. Moreover, treatment of anti-TNFα in alcoholic hepatitis patients, based on extensive data supporting the pathogenic role of this cytokine and initial exciting preliminary outcome^13^, showed a paradoxical increase of patient mortality due to infections^14^.

These despairing circumstances, corroborated with new insights into differences between human and mouse immune system^15,16^, prompted our hypothesis that only limited pathophysiological pathways may be shared between human ALD and different murine models of chronic alcohol consumption routinely used in preclinical studies. The primary goal of our study was to identify the degree of cellular, immunological, and transcriptional similarity profiles between two of the most common murine models of chronic alcohol exposure; secondarily, we compared both murine models at the transcriptional level with the most severe form of human ALD, alcoholic hepatitis, to depict if common pathways are present in these early models of alcohol hepatic toxicity and alcoholic hepatitis.

**Figure 1:**
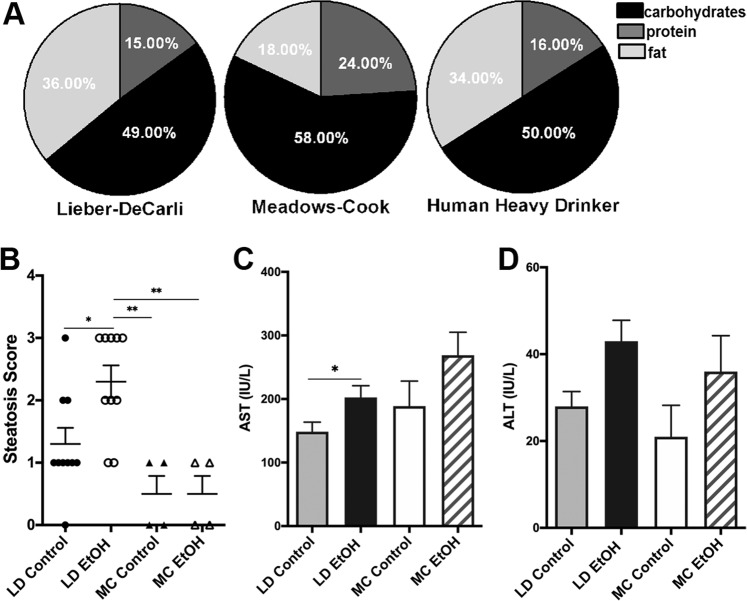
Increased liver injury and steatosis in LD mice. **(A)** Macronutrients composition of LD diet control, chow food diet used in MC model and the usual human heavy drinker alcoholic. **(B–D)** Mice were subject to LD control (LD Control), LD ethanol (LD EtOH), regular chow diet (MC Control) and chronic alcohol exposure as per MC model (MC EtOH). Steatosis severity score **(B)** assessed after hematoxylin and eosine staining of liver sections and confirmed by Oil Red O in mice on LD diet and MC model, without (control) or with alcohol (EtOH). The data represent the mean ± SEM, *p < 0.05, **p < 0.01 (2-way ANOVA with Tukey’s test, n = 10 mice LD group, n = 4 mice MC group). Hepatocyte injury measured by activity of plasma ALT **(C)** and AST **(D)** in mice fed without or with alcohol in LD and MC models. The data are shown as the means ± SEM, *p < 0.05 (2-way ANOVA with Tukey’s test, n = 10 mice LD group, n = 10 mice MC group).

There are two frequently used and accepted murine models of chronic alcohol exposure: Meadows-Cook diet and isocaloric Lieber-DeCarli diet. Meadows-Cook diet (MC) model mice are receiving chow food ad libitum alcohol. Exposure to alcohol consists of a two-week long ramping phase from 0% EtOH to 20% EtOH in water (v/v)^17,18^. Mice are held on this “alcohol-in-water” model for anywhere from 4–16 weeks, with the majority of the studies setting the final readouts after 3 months of alcohol exposure^17–20^. This model was developed to gain an immunological perspective of the specific effects of alcohol, while avoiding any confounding features that may come with changes of the diet composition. On the other hand, mice on Lieber-DeCarli (LD) which is an isocaloric liquid diet, go through a similar ramping phase from 0% to 3.395% EtOH (w/v)^21^. To explore chronic alcohol effects, duration of exposure to LD diet is also variable between 25 days to 8 weeks, with an average around one month^22–29^. This model was created to “enhance” the mice ALD phenotype to be similar to the human ALD patient phenotype and to avoid confounding effects of differences in caloric content of oral intake of alcohol consumption. While mice on the MC model consume a solid diet rich in carbohydrates (58%), mice on liquid LD diet similar with overall human alcoholics, have an enriched intake of lipids and low intake of proteins (Fig. 1A)^30^. Overall, it is unclear how much of the described phenotype (cellular, immunological, and transcriptional) observed in these models are related with the specific diet (liquid/high fat in LD versus solid/chow diet in MC), alcohol, or a combination of both. In addition, it is unknown whether these models share any pathological loops present in the most clinically significant form of ALD, alcoholic hepatitis.

## Results

### LD mice have increased early liver injury and steatosis

Female C57BL/6 6 weeks old mice received LD for 4 weeks with or without alcohol or were exposed to alcohol for 12 weeks as per MC model. The duration of exposure to alcohol for the MC model was extended to 12 weeks instead of 4 weeks based on the previous literature data supporting the presence of clear cut alcohol related immunological effects after this time period^17^, as well as our own validation (data not shown). Ethanol trends to increase the liver/body weight ratio in both models (Supplementary Figure 1A) but none reached statistical significance in spite of significant amount of alcohol consumed by mice in both models (Supplementary Figure 1B). Mice in LD model drank statistically significant more alcohol than in MC model. Despite longer duration of exposure to alcohol, as reported in the literature^31,32^, only alcohol added to LD diet increases hepatic steatosis severity estimated by pathological score (Fig. 1B) while no significant difference is grossly noted by histology in mice exposed to alcohol in MC model (Supplementary Figs. 2 and 3).

On the other hand, level of blood Aspartate Aminotransferase (AST) but Alanine Aminotransferase (ALT) activity showed minimal but statistically significant hepatocellular injury after 4 weeks in LD mice. In spite of an extended period of alcohol exposure of 12 weeks compared with LD diet only a trend toward more liver injury induced by alcohol is noted in MC model (Fig. 1C,D). Earlier development of steatosis and liver injury in LD mice compared with MC mice points toward an additive or synergistic effect of alcohol and fat enriched liquid diet on the steatosis development and liver injury.

### Hepatic immune cells are differentially and specifically affected by LD diet compared with MC model

The hallmark of progressive alcoholic liver injury in humans is the presence of inflammation^33^. In mice, there is no significant associated inflammation identifiable with H&E staining after chronic alcohol treatment in both murine models (data not shown). However, the sensitivity to assess changes in the hepatic immune cell numbers and compositions by this classical methodology is low and requires major changes in the immune cell numbers. Therefore, to identify early changes after alcohol exposure we thoroughly investigated the inflammatory infiltrate by polychromatic flow cytometry after isolation of immune cells from murine livers exposed to alcohol in LD and MC models using a well validated gating strategy^34^.

**Figure 2:**
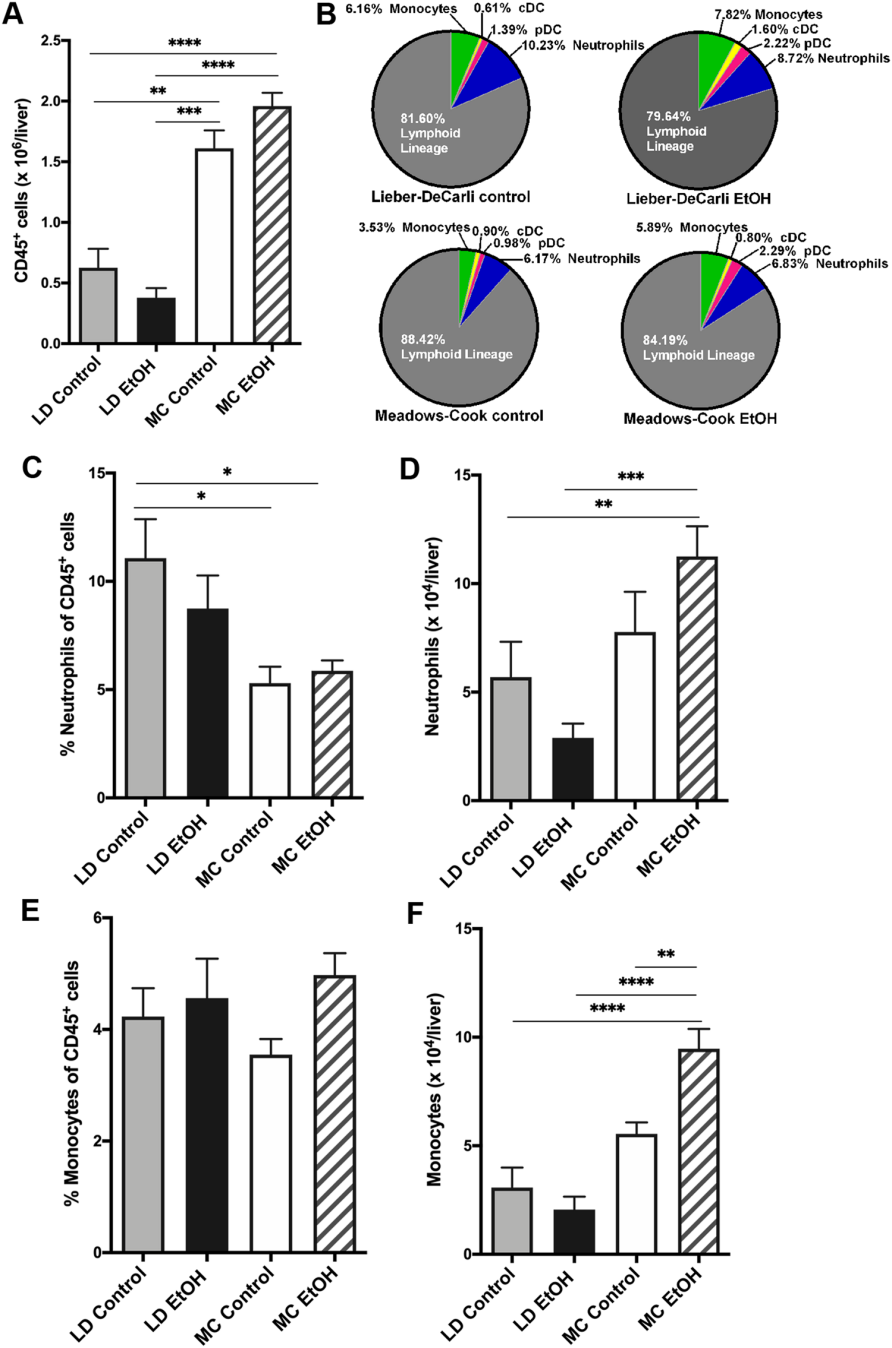
Comparison of innate hepatic immune cells between murine models of chronic alcohol exposure. Mice were subject to LD control (LD Control), LD ethanol (LD EtOH), regular chow diet (MC Control) and chronic alcohol exposure as per MC model (MC EtOH). Intrahepatic leukocytes population were isolated and stained for polychromatic flow cytometry. **(A)** The amount of CD45^+^ cells/liver were quantified in all four groups. The data represent the mean ± SEM, **p < 0.01; ***p < 0.001; ****p < 0.0001 (2-way ANOVA with Tukey’s test, n = 10 mice LD group, n = 16–22 mice MC group). **(B)** The composition of overall lymphoid **(**NK, NKT, B and T cells) and main innate immune cells (monocytes, neutrophils and dendritic cells). The frequencies and absolute cell count per liver of neutrophils **(C,D**) and monocytes **(E,F)** were calculated. The data represent the mean ± SEM, *p < 0.05; **p < 0.01; ***p < 0.001; ****p < 0.0001 (2-way ANOVA with Tukey’s test, n = 10 mice LD group, n = 16–22 mice MC group).

Surprisingly, mice on LD (both groups, with or without alcohol treatment) have less than half of the amount of CD45^+^ cells per liver compared with MC control mice receiving chow diet (Fig. 2A). Secondly, addition of alcohol to LD diet trends toward a paradoxically decreased amount of CD45^+^ cells per liver, as opposed to MC model where liver has more CD45^+^ cells induced after alcohol exposure for 12 weeks without statistical significance (Fig. 2A). When analyzed by composition, the main myeloid cells, neutrophils and monocytes, represents only a minority of immune cells from all hepatic CD45^+^ cells in both murine models; the most frequent cells present in the inflammatory infiltrate, independent of the group, belongs to the lymphoid lineage identified as CD3/CD19/NK1.1 positive cells (Fig. 2B). Despite this strikingly high lymphoid predominance of hepatic inflammatory infiltrate in murine models of alcohol consumption, there are significant differences of frequencies and absolute numbers of specific hepatic myeloid cells specific for each murine model and presence or absence of alcohol exposure.

Neutrophils and monocytes are considered critical for ALD pathogenesis and one of the hallmarks of recent alcohol exposure and alcoholic liver injury in human is the presence of neutrophilic inflammation^33–36^. Regarding neutrophils, only in alcohol-treated MC mice there is a trend, not reaching significance, toward an increase of neutrophils per liver. Paradoxically, there is a trend toward decreasing numbers of neutrophils per liver in mice receiving alcohol LD diet for 4 weeks (Fig. 2C,D). Next, only MC EtOH mice show a significant increase of monocyte numbers and frequencies compared with MC control while no changes are found by addition of alcohol to LD diet (Fig. 2E,F). Moreover, in spite of similar frequencies of monocytes in mice on LD diets (with or without alcohol) and mice on chow diets, numbers of monocytes per liver is reduced to approximately 50% in LD control compared with MC control, correlated with the overall decrease in the numbers of CD45^+^ cells per liver observed in LD groups, independent of the alcohol exposure.

These data support the concept that in spite of early histological changes of early alcoholic liver injury by LD model (e.g. steatosis) similar with human counterpart, MC model has more cellular immunological changes similar with human alcoholic liver injury that is not reproduced by exposure of alcohol in LD diet alone at the cellular level.

### Immune cells’ recruitment transcriptome signature is specific for murine models of chronic alcohol exposure and human alcoholic hepatitis

Our previous analysis of immune cell recruitment in alcohol exposure models showed specific hepatic immune cell numbers and compositions induced by each model and presence of alcohol. To explore whether there are specific or common mechanisms responsible for differential immune cells recruitment in these two models, we measured the level of transcripts involved in the recruitment of hepatic immune cells by real-time quantitative PCR (RT-qPCR). Recruitment of the cells in the liver is dependent on the chemokine gradient as well as the adhesion molecules present on the endothelium^37–39^. Initially, we assessed the mRNA transcripts involved in the development of hepatic chemokine gradient and mRNA for endothelial adhesion molecules. Secondly, we took advantage of published transcriptome data from the most severe form of ALD^40^ and compared with these “immune cell recruitment signatures” obtained from our murine models to investigate similarities with human alcoholic liver injury.

**Figure 3:**
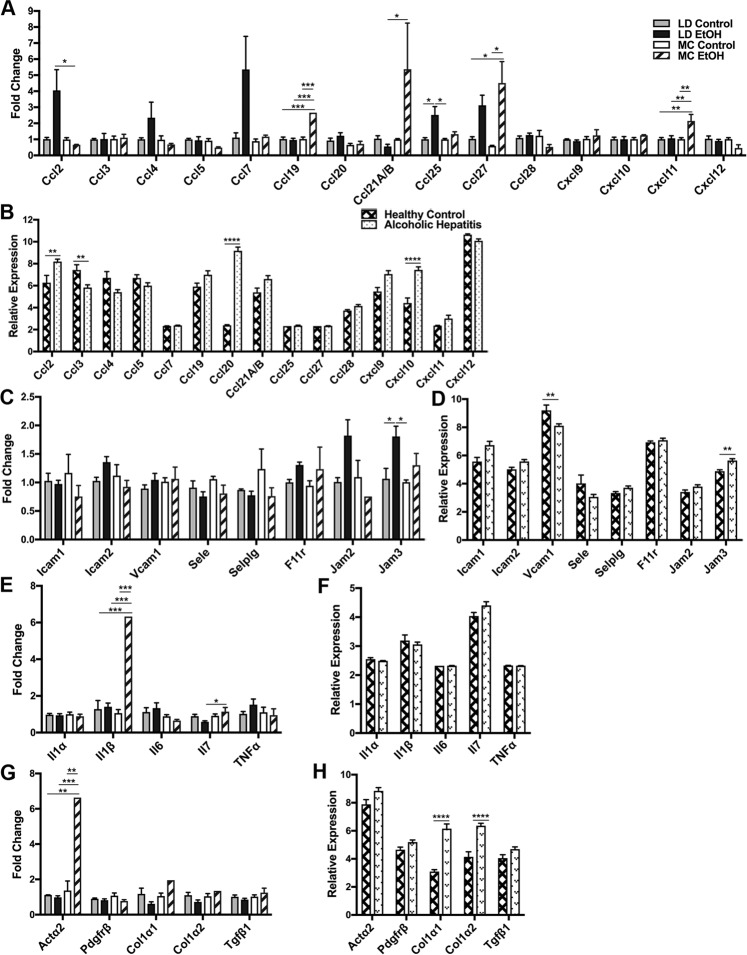
Cross-species comparison of transcriptome signature between murine models of chronic alcohol exposure and human alcoholic hepatitis. Mice were subject to LD control (LD Control), LD ethanol (LD EtOH), regular chow diet (MC Control) and chronic alcohol exposure as per MC model (MC EtOH). Hepatic total mRNA was extracted and analyzed by qRT-PCR for expression changes if chemokines involved in recruitment of neutrophils and monocytes **(A)**, endothelial adhesion molecules **(C)**, main cytokines involved in ALD pathogenesis **(E)**, and fibrogenic markers **(G)**. The comparative C^t^ method (ΔΔC^t^) was used to analyze RT-qPCR data. Fold change was calculated by using the geometric means of the housekeeping genes (Actb and B2m) followed by calculation of ΔΔC^t^ value. The data are shown as the means ± SEM, *p < 0.05; **p < 0.01; ***p < 0.001; ****p < 0.0001 (2-way ANOVA with Tukey’s test, n = 4–8 mice LD group, n = 3–6 mice MC group), Relative expression of similar markers (**B,D,F,H**) were calculated using the dataset from the public domain (GSE28619). The data are shown as the means ± SEM, **p < 0.01; ****p < 0.0001 (Adjusted p-value analyzed by multiple t test with the Holm-Sidak method, n = 7 Healthy Control group, n = 15 Alcoholic Hepatitis group).

First of all, looking from the perspective of the type of target cells recruited by chemokines, in murine models, only Ccl27 mRNA (involved in memory T cells recruitment) is up-regulated by alcohol in both MC and LD models, however without reaching statistical significance in 4 weeks LD model (Fig. 3A). Ccl19 mRNA (involved in recruitment of dendritic cells, antigen-engaged B cells and central memory T cells) and Cxcl11 (chemotactic for activated T cells) are upregulated in MC EtOH mice. For comparison in human alcoholic hepatic samples, there are statistically significant up-regulations of a wide variety of chemokines mRNAs involved in recruitment of myeloid (Ccl2), mixed myeloid/lymphoid (Ccl20, Cxcl10), and lymphoid (Ccl21) cells but none these changes are present in murine models (Fig. 3B). Secondly, looking into endothelial adhesion molecules controlling the immune cells’ recruitment, Jam3 mRNA is increased similarly in both human alcoholic liver and LD EtOH mice (Fig. 3C,D). Moreover, in spite of some cellular immune differences identified by flow cytometry, there are not statistically significant changes of endothelial adhesion molecules’ mRNA in MC model (Fig. 3C). Finally, we compared if differential hepatic immune cell numbers and compositions have an impact on transcripts featuring hepatic cytokine milieu (Il1α, Il1β, Il6, Il7, Tnfα) and fibrogenesis (Acta2, Pdgfrβ, Col1α1, Col1α2, TGfβ1). In mice, Il1β and Il7 transcripts were up-regulated significantly by alcohol in MC model (Fig. 3E) while only Actα2 was the single classical fibrogenic gene statistically up-regulated by chronic alcohol exposure in MC models (Fig. 3G). For comparison, collagen transcripts are highly up-regulated in the human alcoholic liver disease dataset and this is concordant with histological samples documenting cirrhosis in 60% of cases^40^. Remarkably, based on the data present in the aforementioned GEO public dataset, many of the cytokine transcripts are completely unchanged including Tnfα transcript (Fig. 3F,H).

### RNA-seq signature in murine models

Our comparative analysis initially focused on immune cell recruitment mechanisms between these two murine models of alcohol exposure showed a very restricted number of pathways induced by alcohol. To evaluate whether these two models have common or distinctive gene signatures apart from the immune system we performed RNA sequencing of whole liver samples.

**Figure 4:**
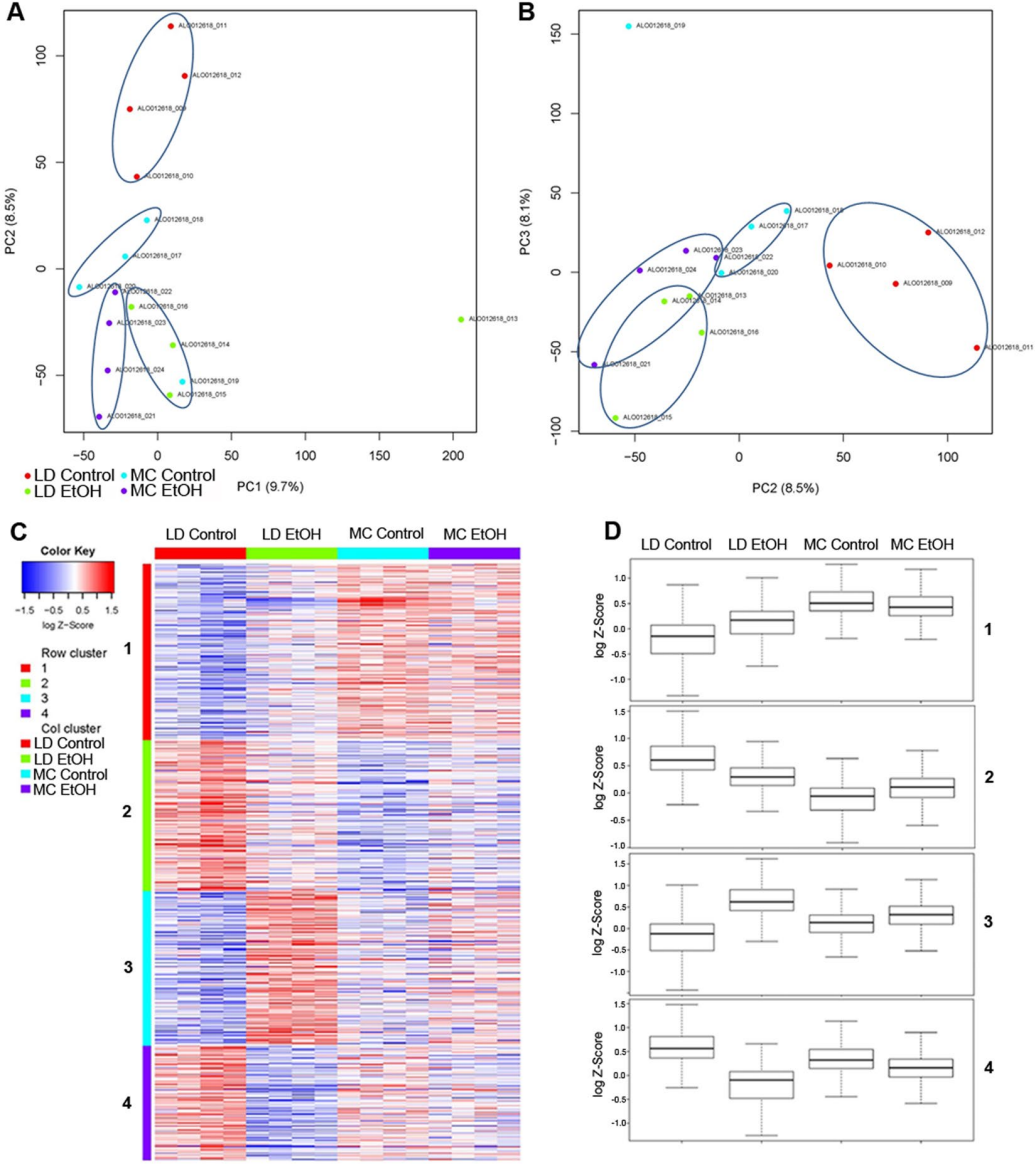
RNA-seq analysis of ethanol induced changes by alcohol in LD and MC model. RNA-seq of hepatic tissue of mice subjected to LD control (LD Control), LD ethanol (LD EtOH), regular chow diet (MC Control) and chronic alcohol exposure as per MC model (MC EtOH) was performed. **(A,B)** Principal component analysis of all 4 groups where PC1 reflected the effect of alcohol, PC2 reflected the effect of the diet, and PC3 the combination. **(C)** Heat map and **(D)** boxplots after hierarchical cluster analysis of differential expressed genes (DEG) between the 4 groups. The heatmap was constructed in R using heatmap.2 function in the gplots package. Gene expression magnitude is represented by the color ranging from high expression (red) to low expression (blue). The boxplots for each cluster and the respective condition were generated to visualize quantitative patterns differences in the cluster patterns.

Overall, principal component analysis (PCA) plots clearly identify distinct clusters of RNA expression patterns of LD control mice (LD Control) compared to their alcohol counterparts (LD EtOH), as well as compared to Meadows-Cook treated and untreated mice (MC EtOH, MC Control) (Fig. 4A,B). Clearly, addition of alcohol in isocaloric models (LD EtOH group) displayed a more significant and distinctive RNA-seq signature versus LD Control than MC EtOH versus MC Control.

Supervised gene clustering of all four experimental groups identified 4 distinct patterns of gene signature changes in relationship with the type of model/diet and presence of alcohol (Fig. 4C,D). The first two clusters of genes are composed from genes that are discordantly affected by type of murine model (MC versus LD) and presence of alcohol exposure. First cluster includes genes that are up-regulated in LD EtOH and down-regulated in MC EtOH when compared with control. The second cluster is composed of genes down-regulated in LD EtOH and up-regulated in MC EtOH. The last two clusters of genes are composed of genes affected by addition of alcohol in similar direction (concordant) in both models either being up-regulated (Cluster 3) or down-regulated (Cluster 4).

**Figure 5:**
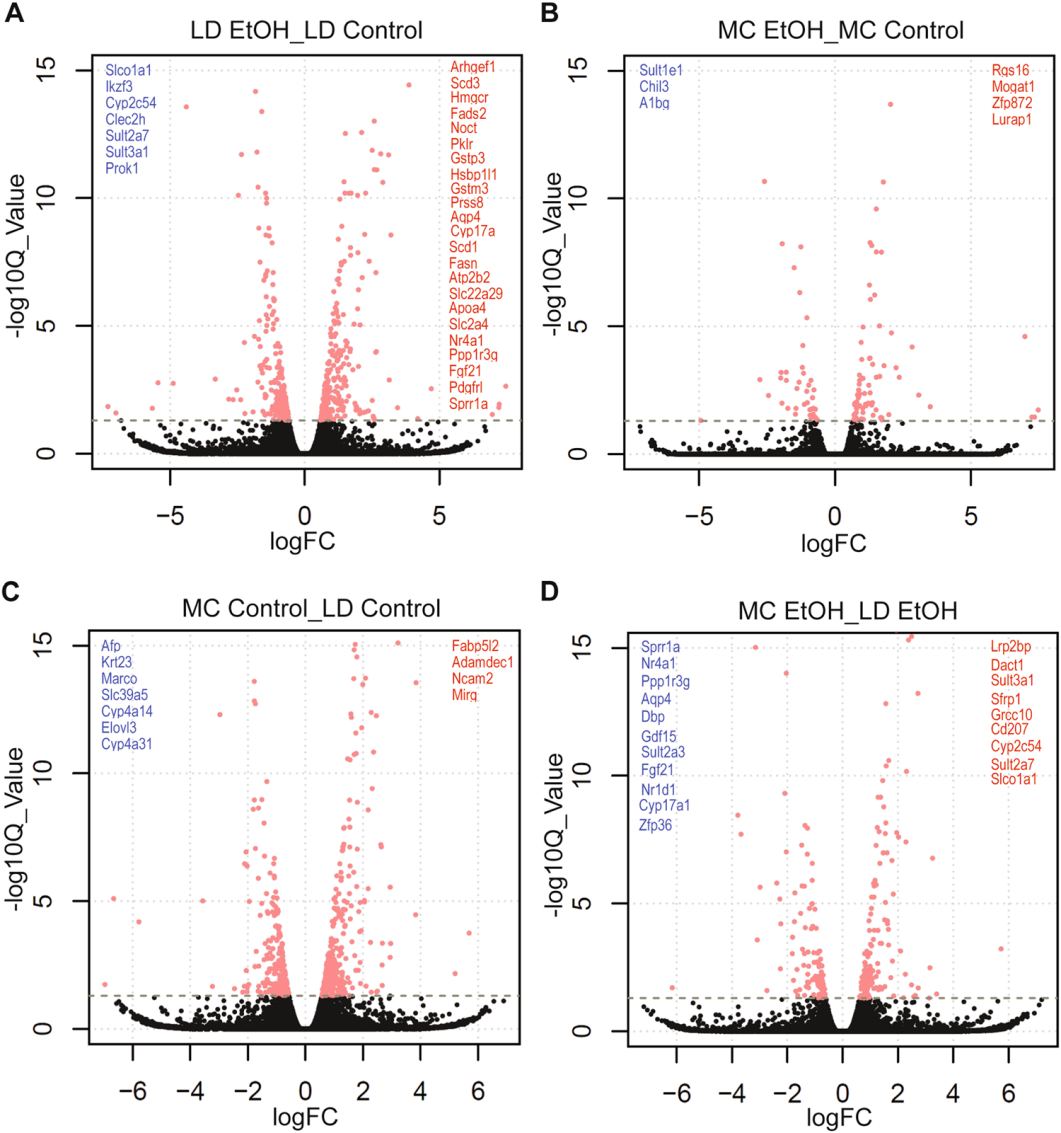
Volcano plots illustrating the DEGs in LD and MC model. Differential gene expression induced by alcohol **(A,B)** and diet type **(C,D)** in mice subjected to LD control (LD Control), LD ethanol (LD EtOH), regular chow diet (MC Control) and chronic alcohol exposure as per MC model (MC EtOH). Volcano plots were generated using R (version 3.5.3) to visualize significance and fold changes of DEG obtained from RNA-seq data. In the volcano plots, X-axes: log2 FC (log fold changes of normalized gene expression in the 1^st^ group compared with the 2^nd^ group; Y-axes: −log10 Q-value. Differentially over-expressed genes in the 1^st^ comparison group are marked in red, while down-regulated genes are in blue, with FC >2 fold and FDR >0.05.

Because we observed specific gene signatures in each of the studied groups, we asked what specific genes are affected by alcohol or diet type in either of murine models. As predicted by PCA analysis, in LD model addition of alcohol results in more significant number of differential expressed genes (DEGs) compared with MC models. When using the cut-off of change (≥2 fold), *q*-value (FDR ≤0.05), and include only the genes coding protein with known function we observed: (1) only 4 genes are specifically up-regulated in MC EtOH compared with 23 in LD EtOH and (2) only 3 specifically down-regulated on MC EtOH compared with 7 in LD EtOH (Fig. 5A,B; Supplementary Table 1). Addition of alcohol to LD diet (LD EtOH) resulted in up-regulation of 5 genes involved in lipid metabolism as fatty acid synthesis (*Fasn*), conversion of saturated fatty acids into unsaturated fatty acids (*Scd3, Scd1*), cholesterol biosynthesis (*Hmgcr*) as well as chilomicrones transporter gene (*Apoa4*). In constrat, from all 4 genes specifically up-regulated by alcohol in MC model, only one is involved in lipid metabolism: *Mogat1*.

In the absence of alcohol, specific to MC model when compared with LD, up-regulation of 4 genes and down-regulation of 7 genes are revealed; among these 7 specific genes down-regulated by MC two belong to cytochromes families (*Cyp4α14, Cyp4α31*). Whereas, in the presence of alcohol, MC specific gene down-regulation is observed for 11 genes while 9 genes are up-regulated (Fig. 5C,D; Supplementary Table 1) implying the existence of diet specific mechanisms activated by alcohol in these 2 models. Of note, 6 genes (*Sprr1α, Nr4α1, Ppp1r3g, Aqp4, Fgf21, Cyp17α1*) are commonly up-regulated; 4 genes (*Sult3α1, Cyp2c54, Sult2α7, Slcoα1*) are down-regulated by alcohol in LD EtOH versus LD control and also in LD EtOH versus MC EtOH, supporting the concept that LD model uncovers additional diet-specific pathways not observed in MC model.

**Figure 6:**
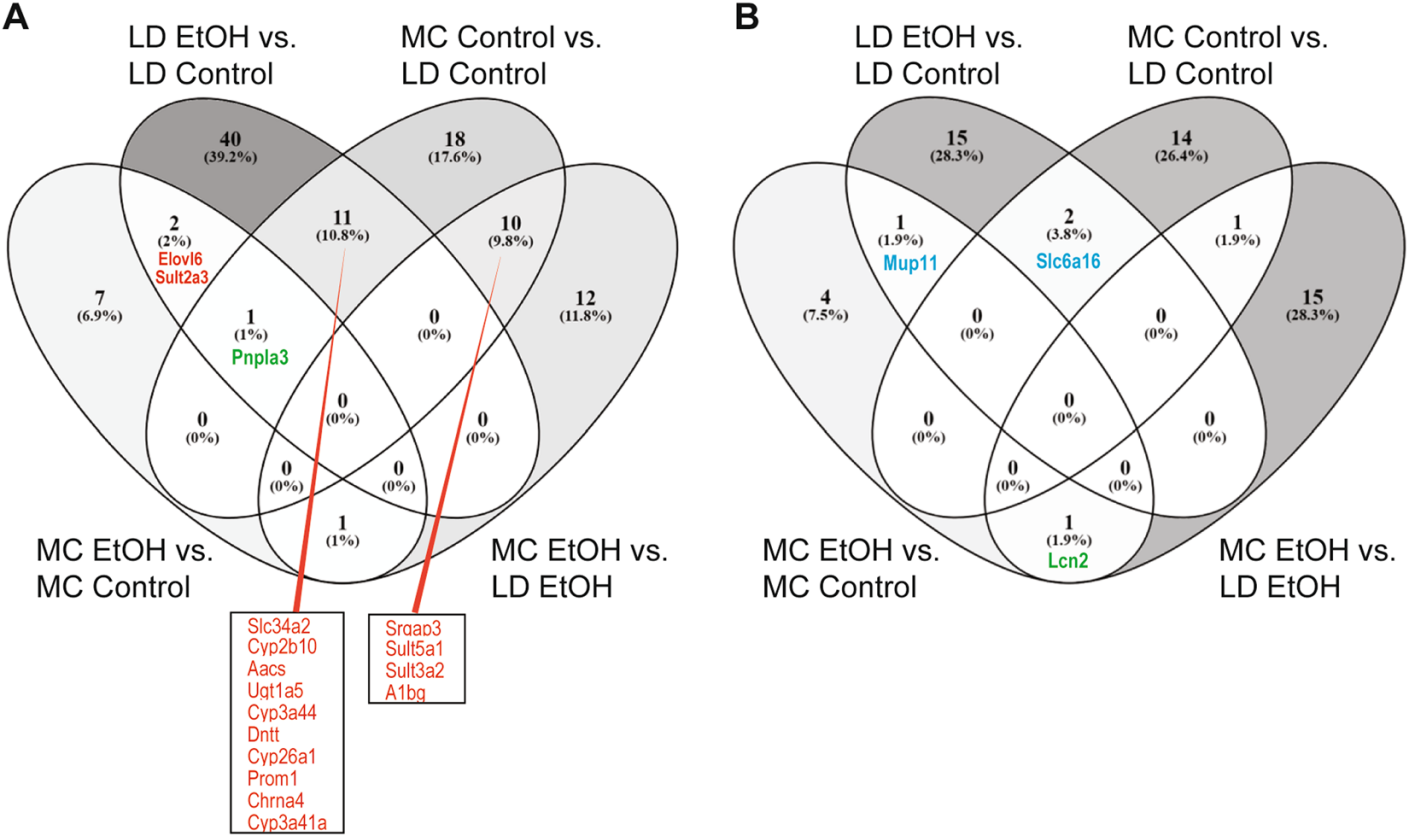
Venn diagrams of DEGs in LD and MC model. RNA-seq of hepatic tissue of mice subjected to LD control (LD Control), LD ethanol (LD EtOH), regular chow diet (MC Control) and chronic alcohol exposure as per MC model (MC EtOH) was performed. Venn diagram showing the overlap of commonly **(A)** up-regulated and **(B)** down-regulated DEGs between mice receiving LD control, LD EtOH, MC Control and MC EtOH.

Next we performed the four-way comparison of shared DEGs by all 4 groups (Fig. 6A,B; Supplementary Table 2) studied in our labs. There is only one common gene down-regulated (*Mup11*) and 2 genes (*Elovl6* and *Sult2α3*) up-regulated by alcohol independent of the type of the diet used. More importantly, *Pnplα3* gene is the single DEG that is down-regulated by the LD diet (MC Control versus LD Control) and up-regulated in both models of alcohol exposure (MC EtOH versus MC Control; LD EtOH versus LD Control) while *Lcn2* gene is the single gene that is down-regulated by alcohol in MC model but up-regulated in LD model (MC EtOH versus MC control and MC EtOH versus LD EtOH).

To gain further insight and validation into the specific biological functions of DEGs, we performed GO analysis by querying how the addition of alcohol in both models affects gene expression clustered by a specific biological function.

**Figure 7:**
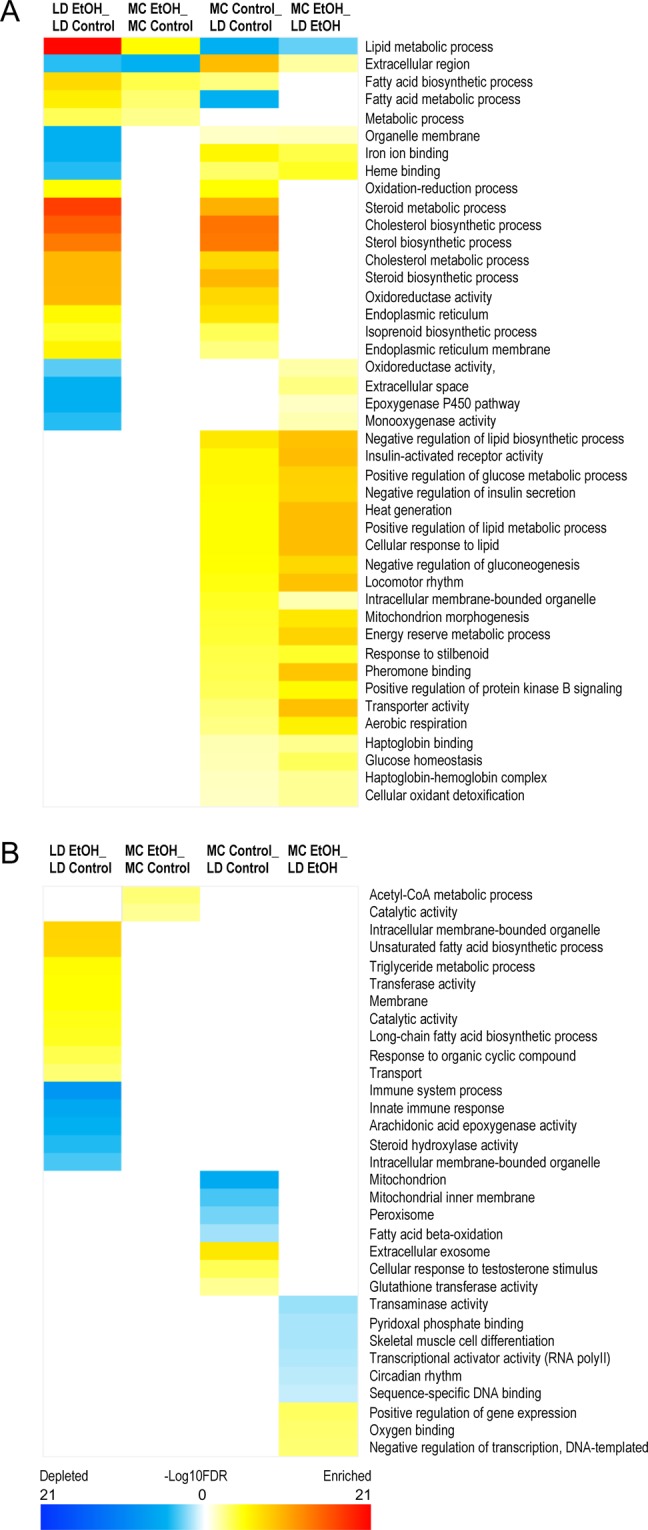
Heat maps of gene ontology (GO) analysis for DEGs in LD and MC model. RNA-seq of hepatic tissue of mice subjected to LD control (LD Control), LD ethanol (LD EtOH), regular chow diet (MC Control) and chronic alcohol exposure as per MC model (MC EtOH) was performed. Heat maps of GO term of shared **(A)** and group-specific **(B)** DEGs induced by alcohol were obtained by color quantification of −log10 FDR (red: up-regulated, blue: down-regulated).

Our observation of the analysis was that using similar very stringent cut-off for *p*-values and FDR (<0.05) there are only 5 similar pathways affected by exposure of alcohol in these 2 models (MC EtOH/MC Control versus LD EtOH/LD Control) (Fig. 7A). While genes involved in processes of lipid and fatty acid metabolism are up-regulated, the genes involved extracellular biology are down-regulated. Specific to each model, we observed an enrichment of genes involved in metabolism of Acetyl-CoA that is restricted to MC EtOH group while down-regulation of genes involved in innate immunity is highly altered by exposure of alcohol in LD diet (Fig. 7B).

## Discussion

A variety of murine models of alcohol consumptions are frequently used in preclinical investigations to explore the mechanisms responsible of the immunological effects of alcohol and subsequently used to test novel treatments of ALD^41,42^.

We aimed to assess hepatic phenotype profiles at cellular immune, transcript, and histological levels in the two most common models of chronic alcohol consumption: MC model and isocaloric LD diet. While MC model mice are receiving regular chow diet, LD diet mice were fed with a liquid diet enriched in lipids. This study documented that interpretation of histological, cellular immune and transcriptome data obtained in murine models should strongly consider the type of model used. Moreover, when we compared gene expression similarities between these murine models of alcohol exposure and human alcoholic hepatitis data from the public domain we found very limited specific shared changes.

Our first observation was that at the histological level, there is more hepatic steatosis in mice on LD with or without alcohol for only 4 weeks compared with the mice exposed on standard 12 weeks MC. This is anticipated considering the contribution of fat to overall caloric intake and supports the concept of augmentation of steatosis by combination of dietary fat and alcohol. Histological effect of LD diet seems to have some correlation with the amount of injury. Although there is a trend toward an increase liver injury assessed by liver enzymes in mice in MC model, addition of alcohol increases AST compared with paired control only in LD diet.

Chronic models of alcohol consumption in mice are characterized by minimal to none inflammation^42^. As expected, in both models histological grading of inflammation does not depict any significant effect of alcohol when compared with control mice (overall median inflammation severity less than 1). Absence of any alcohol specific effect on overall number of hematopoietic derived cells in the liver is confirmed after the isolations and quantification of total hepatic CD45^+^ cells in both models.

Completely unexpectedly, there were three times less hepatic CD45^+^ cells in mice on LD diet when compared with the MC model. Interestingly, it is anticipated to have a significant number of myeloid cells in the murine liver, when we looked into the composition of the hepatic CD45^+^ cells in MC and LD diet, around 80% of all CD45^+^ cells belong to the lymphoid lineage (NK cells, B cells, CD3^+^ cells).

Neutrophils are frequently encountered as a part of histological features of alcohol liver injury in humans^36^. Alcohol does not significantly increase the number of hepatic neutrophils in the liver of mice on either diet, in spite of an increasing trend in the MC model. Furthermore, mice on the LD diet (both control and alcohol) have less numbers of neutrophils compared with MC mice. Moreover, alcohol exposure for 4 weeks in this model (LD EtOH) is associated with an unexpected trend toward a decrease of the number of neutrophils compared to control (LD Control). This suppressive effect of LD diet on neutrophil populations can be explained and additionally explored in the future by potentially complex mechanisms involving neutrophilic bone marrow development and egress, endothelial adhesion molecules dependent, and cytokine gradient hepatic recruitment, or decreased survival of neutrophils in the pro-inflammatory hepatic environment with steatosis.

Monocytes are another type of innate cells considered to be important in pathogenesis of alcoholic liver injury in humans and potentially cellular targets for new drugs^43–45^. Similar with the neutrophils, LD has a suppressive effect on absolute monocyte counts, independent of the alcohol exposure. Interestingly enough, alcohol has a specific effect on monocytes frequency and amount only in the liver of mice in MC model. This difference clearly supports the concept that MC model may be used to explore alcohol-specific monocyte effect targeted drugs.

Similar with cellular immunological changes, limited amount of common shared abnormalities are observed between the two models of murine alcohol exposure at the transcript levels. In contrast to human data where Ccl2, Ccl3, Ccl20 and Cxcl10 transcripts were significantly changed in alcoholic hepatitis compared to healthy control, none of these statistically significant changes were found in the murine models. A possible explanation is that murine models have only limited exposure to alcohol when compared with humans while the other explanation is that severity of the inflammation is only limited and very early upon alcohol toxicity in murine models. The predominant increase in the lymphocyte recruitment chemokine gradient in mice compared with myeloid in humans raises the question of species specificity of the mechanism involved in alcohol induced inflammation. This comparison of two murine models of chronic alcohol exposure with human data present in public domain has to take into account also the limitation of severity of liver disease, sex distribution, and age of patients included in the study. The human samples used for generation of transcriptomic data belongs to adult patients (mean of 49 years old), more than half males, majority with severe alcoholic hepatitis (77.5%) and cirrhosis (60%), with alcohol intake of 107 g/day^40^ while in our murine studies samples belong to relatively young female mice, with mild liver tissue injury (only steatosis) and average of alcohol consumption between 16–21 g/Kg/day.

Consistent with our findings from RT-qPCR of liver tissue, the whole hepatic RNA-seq analysis confirmed that only limited and restricted number of genes were changed by alcohol exposure in both models.

As a result, 2 different specific gene signatures of DEGs affected by alcohol exposure in these models (cluster 1 and 2 of DEGs in Fig. 4C,D) support the concept that each models should be considered unique and highly dependent on the qualities of the diet (amount of fat, physical state).

The uniqueness of interaction between alcohol and diet is reflected on our analysis of specific and common DEGs affected by introduction of alcohol in these two models. Not surprisingly, the highly fat enriched LD model is characterized by a specific up-regulation of lipid metabolism gene signature^46^ as well as glutathione metabolism while MC model has a less discreet effect on genes involved in this process. This close interaction between the diet and alcohol in mouse model is supported by the well-validated observation of relationship between the obesity and ALD progression^47^.

In spite of limited common pathways affected by alcohol in these two models both of the models validate *pnlpα3* as a major and single gene commonly changed by alcohol, fat, and alcohol and fat combination when compared with their respective controls. This is not a surprise considering this gene is well known to increase clinical setting susceptibility not only to non-alcoholic steatohepatitis (NASH)^48^ but also to ALD^49,50^. On the other hand, the limited number of genes similarly affected by alcohol in both models raises the question of murine models diversity and mechanistic diversity of ALD in humans based on the environmental factors.

What is especially exciting is that our DEG analysis provides hints for potential biomarkers that may help differentially diagnosis between NASH, ASH, and combination of NASH and ASH. All these entities are more and more intricate in the clinical practice due to an increase of obesity prevalence in the general population, having similar histological features. Oftentimes mild/moderate ASH are difficult to differentiate from NASH with an interobserver variability in recognizing histological features of ASH that is limited and barely reaching 50%^51^. Based on our analysis, Lcn2 (Lipocalin-2), well known to be affected in murine models using LD model^52,53^, is actually down-regulated by alcohol in MC model and has potential to make the difference between plain ASH (MC EtOH equivalent) and ASH + NASH (LD EtOH equivalent). Moreover, our DEG analysis, suggested two genes, *Elovl6 and Sult2a3*, changed by exposure of alcohol in both diets (diet-independent) that can potentially be used to design alcohol specific, diet independent, drug targeting but also potential diet independent biomarkers of alcohol toxicity.

Previous published work showed that alcohol effects on immune system are dependent of the duration and dose of alcohol exposure as well as gender and age of mice, so our present comparative work between these two most common murine chronic models used to study the effect of alcohol has some limitations^19,54,55^.

First of all, our study is limited to the comparison of only two most common utilized durations of chronic alcohol exposure of 4 weeks for LD diet and 12 weeks of MC model so two different magnitudes of alcohol exposure^22,24–29^. However, it is pretty well known that alcohol effects on specific immune cells and tissue injury are highly dependent of alcohol exposure time^56–59^. For example, alcohol decreases NK cell proliferation at 2 weeks while increases splenic NK cell pool at 3 months^19^.

Secondly, chronic alcohol drinking increases inflammatory transcriptomic profile following LPS stimulation in peripheral blood mononuclear cells in dose dependent manner^54^. A confounding factor in our study is that the amount of alcohol per day consumed in LD model was higher compared with MC model in spite of more extended time of alcohol exposure in MC mice. For this reason, a comparison of 4 weeks MC model with 4 weeks LD diet potentially decreases the duration of alcohol exposure effect but increases more the differences between the amount of alcohol consumed by the mice in each model.

Third of all, to increase the sensitivity of our analysis we used in our experiments only female mice as is pretty well known that the effect of alcohol is sex dependent in mice and humans^55,60–64^. Actually, our ongoing current work in the lab is focused on specific cellular immune effects present only in females and not observed in male mice (data not presented, manuscript in preparation).

Last and not least, in order to replicate the most common already published protocols on MC and LD diet, the age of the mice when they start to be exposed to alcohol is pretty young at age of 6 weeks when immune system is still developing. We are very aware of this limitation, so our analysis of the hepatic immune cells is limited only to innate immunity component that is relatively well developed at 6 weeks of age, less age dependent and with a more characterized role in alcoholic liver disease pathogenesis.

In summary our study identifies a very restricted common pathways present in the two most used murine models of chronic alcohol exposure. Furthermore, there are specific cellular and immunological mechanisms highly specific for each model, and some hepatic transcriptome changes present in humans are never seen in murine models in spite of some histological similarities. These data support the concept of the diet specific effect of alcohol effect on the liver; this may be related with the obvious fat content differences but also raises the questions of species-specific mechanisms of alcohol induced organ damage. Whether our observations are the effect of liquid versus solid food (“dynamic action of food state”) or only related with fat-content alone, is at this time matter of additional future investigations. Either way, preclinical testing of targeting alcohol hepatotoxicity effects should consider the model used, followed by a mandatory validation of abnormal pathways present in human targeted organ, and interpreted in the context of highly diet-dependent mechanistic diversity of human and murine alcohol induced pathophysiology.

## Methods

### Mice

Six weeks old female C57BL/6 mice (Jackson Labs) were placed on 2 different murine models of chronic alcohol exposure: isocaloric Lieber-DeCarli diet (LD)and Meadows-Cook diet (MC). For LD, mice received either an EtOH-containing diet (n = 4–10) or an isocaloric pair-fed control diet (n = 4–10) for 4 weeks according to the Lieber-DeCarli regimen (Bio-Serv, Flemington, NJ; #F1258SP, #F1259SP). The amount of EtOH was gradually increased from 1.69% in the first week to 2.55% and 3.395% in the third week (wt/vol). The feeding bottles were replaced daily to measure the amount of diet consumption. On the other hand, MC model mice (n = 3–22) were fed chow food *ad libitum* EtOH for 12 weeks. The mice were ramped up from 0% EtOH to 20% EtOH in water (v/v) with a step-wise increase in EtOH concentration (5%, 10% and 15% for 4 days each and then 20% in the third week). Neither of the mice had free access to water except the MC control. The mice were housed 5 per cage. The detail of the number of mice replicates (n) was mentioned in figure legends. All the experiments involving mice were approved by the Rush University Medical Center (RUMC) Institutional Animal Care & Use Committee (IACUC) and were carried out according to the National Institute of Health (NIH) guidelines.

### Liver histology

Fresh liver tissue was fixed in 10% formalin for 24 hours, embedded in paraffin and stained with standard Hematoxylin and Eosin (H&E). In addition, fresh liver specimen were fixed in Tissue-Tek Optimal Cutting Temperature (OCT) compound (Sakura Inc, CA), processed and stained with Oil Red O. H&E and Oil Red O stained tissue was blindly graded regarding steatosis, inflammation, and ballooning on a score from 0 (least severe) to 3 (most severe) by a hepatologist (CA).

### AST and ALT measurements

The level of ALT and AST was measured with Pointe Scientific liquid ALT (SGPT) and liquid AST (SGOT) reagent sets (#A7526150, #A7561150), according to the manufacturer’s instructions (Pointe Scientific, Canton, MI).

### Flow cytometry

Hepatic leukocyte isolation was performed as previously reported^34^. Briefly, each mouse was perfused through portal vein with 10 mL of cold PBS to remove circulating immune cells present in the liver. Liver was removed and fresh liver tissue was cut on ice into one mm^2^ pieces in one well of a 6-well plate filled with 4 mL of wash buffer (cold PBS containing 2% FBS). The liver pieces were then pushed through 75 μm filters into 50 mL conical of wash buffer. Samples were pelleted and washed with wash buffer. The pellet was resuspended in 3 mL of 70% Percoll and placed underneath 7 mL of 40% Percoll. The solution was spun for 30 minutes at 800 RPM with no brake. Leukocytes at the interface of 70% and 40% Percoll were collected, washed, and resuspended in wash buffer. Leukocytes were stained with fluorochrome conjugated antibodies for CD45, CD3, CD19, NK1.1, Ly6G, PDCA1, MHCII, CD11c, CD11b, and F4/80 (Supplementary Table 3) as well as death/viable cell stain (Fixable Viability Dye eFluor 506, eBioscience, Cat # 65–0866). Flow cytometry was performed using LSRFortessa flow cytometer (BD Biosciences, San Jose, CA). Gating strategy was focused on identification of neutrophils (CD45^+^, CD3/CD19/NK1.1 negative, PDCA-1 negative, CD11b^+^, Ly6G^+^) and monocytes (CD45^+^, CD3/CD19/NK1.1 negative, PDCA-1 negative, CD11b^+^, Ly6G negative, MHCII negative, F4/80^+^).

### RT-qPCR of liver tissue

Liver was flash frozen in liquid nitrogen and then processed by UIC Genomics Core for total mRNA extraction, reverse transcription and quantitative PCR for chemokines involved in hepatic leukocyte recruitment, endothelial adhesion molecules, cytokines previously shown to have a role in pathogenesis of alcoholic liver injury and fibrogenesis markers. Real-time qPCR was performed using TaqMan primers (Supplementary Table 4) on the ViiA 7 Real-Time PCR System (Applied Biosystems, Foster City, CA). Fold change was calculated by using the geometric means of the housekeeping genes (Actb and B2m) to then calculate ΔΔC^t^.

### Human data

Alcoholic hepatitis and healthy subjects’ microarray data was obtained from GEO dataset (GSE28619; https://www.ncbi.nlm.nih.gov/geo/query/acc.cgi?acc=GSE28619)^40^.

### RNA-sequencing of mouse liver samples

Flash frozen liver (n = 16) was processed for RNA-sequencing by UIC Genomics Core as per standard protocol. The library was constructed from 3′ mRNAseq using Lexogen library prep kit (Illumina, San Diego, CA). Samples were sequenced for gene expression using NextSeq500 (Illumina) with a read length of 1 × 75 bases.

The datasets generated during the current study are available in the Gene Expression Omnibus (GEO; http://www.ncbi.nlm.nih.gov/geo/repository in National Center for Biotechnology Information (NCBI).

### Bioinformatics of RNA sequencing

RNA sequencing data was analyzed by UIC Research Informatics Core (University of Illinois at Chicago, Chicago, IL). Raw reads were aligned to the mouse reference genome (mm10) using BWA MEM^65^, which efficiently maps reads with read-through into polyA tails and adapter sequences, as is common with 3′ RNA-seq. The expression level of genes was quantified using FeatureCounts^66^ first as raw read counts, which are suitable for differential expression analyses, and also normalized to reads-per-million for direct comparison between samples. Principle component analysis (PCA) was used to check for biological outliers.

Differential expression statistics (fold-change and p-value) were computed using edgeR^67,68^, on raw expression counts obtained from quantification. EdgeR’s GLM (Generalized Linear Model) was used to perform pairwise analysis between LD Control diet vs LD EtOH, and MC Control vs MC EtOH. EdgeR’s exact test was used for multi group analysis to define the effect of Diet (LD, MC) vs treatment (Control, EtOH). We adjusted p-values for multiple testing using the false discovery rate (FDR) correction of Benjamini and Hochberg^69^. Significant genes were determined based on an FDR threshold of 5% (<0.05).

For clustering and visualization of the data, we identified distinct and robustly separable genomic patterns using a discovery clustering analysis that pairs *k*-means clustering with consensus clustering statistics. We performed *k*-means clustering with ten random initializations on a range of cluster numbers k (2 to 20). For each *k*, the reproducibility of the repeated clustering runs was evaluated using consensus clustering statistics, as outlined by Senbabaoglu and co-workers^70^, choosing the highest number of clusters that yields highly reproducible clusters. This procedure provides the most granular segregation of data heterogeneity, without over-interpreting random patterns in the noise. Using this technique, we identified 4 clusters in the dataset. The heatmap was constructed in R using heatmap.2 function in the gplots package to visualize the different clustering patterns. To visualize quantitative patterns differences in the cluster patterns, we generated boxplot for each cluster and the respective conditions.

Volcano plots were generated using R version 3.5.3 to visualize significance and fold changes of DEG obtained from RNA-seq data. In the volcano plot, log fold changes of normalized gene expression in the first group compared with the second group were used as an x-axis and -log10 Q values were used as a y-axis.

Two interactive Venn diagrams of shared upregulated and downregulated DEG between all 4 studied groups were generated taking into account DEG of at least 2 folds with a q-value (FDR) of less than or equal to 0.05.

Gene Ontology (GO) analysis was applied to analyze the main function of DEGs according to the NCBI Gene Ontology database, which provides key functional classifications for genes^71^. GO categories with FDR equal or less than 0.05 were selected for visualization as heatmaps for shared and specific gene functions affected by addition of alcohol to LC and MC model or the effect of type of diet in the absence of presence of alcohol.

### Statistical analysis

GraphPad Prism version 8.3 (GraphPad Software, Inc. San Diego, CA) was used for all statistical analyses. Multiple *t*-test with the Holm-Sidak method was used to compare two groups, two-way ANOVA followed by Tukey’s multiple comparisons test was used to compare 2 variables in more than 2 groups. Asterisks indicate levels of significance as follows: ^*^p < 0.05; ^**^p < 0.01; ^***^p < 0.001; and ^****^p < 0.0001. Data are presented as means ± standard error of means (SEM).

## Supplementary information

Supplementary Information.
