# Supplementary material for: Restricted immunological and cellular pathways are shared by murine models of chronic alcohol consumption
Source: Sci Rep. 2020 Feb 12;10:2451. doi: 10.1038/s41598-020-59188-9 (PMC7016184; doi:10.1038/s41598-020-59188-9)
Supplement: Supplementary file 1 — Supplementary Information. [file 41598_2020_59188_MOESM1_ESM.pdf]

SUPPLEMENTARY INFORMATION

Restricted immunological and cellular pathways are shared by murine models of chronic alcohol consumption

Alyx Vogle[1], Tongqi Qian[2], Shijia Zhu[2], Elizabeth Burnett[1], Holger Fey[1], Zhibin Zhu[1], Ali Keshavarzian[1], Maliha Shaikh[1], Yujin Hoshida[2], Miran Kim[1], Costica Aloman[1*]

[1]Division of Digestive Diseases and Nutrition, Section of Hepatology, Rush University, Chicago, IL, USA

[2]University of Texas Southwestern Medical Center, Division of Digestive Diseases, Department of Internal Medicine

* indicates corresponding author

Supplementary Figure 1

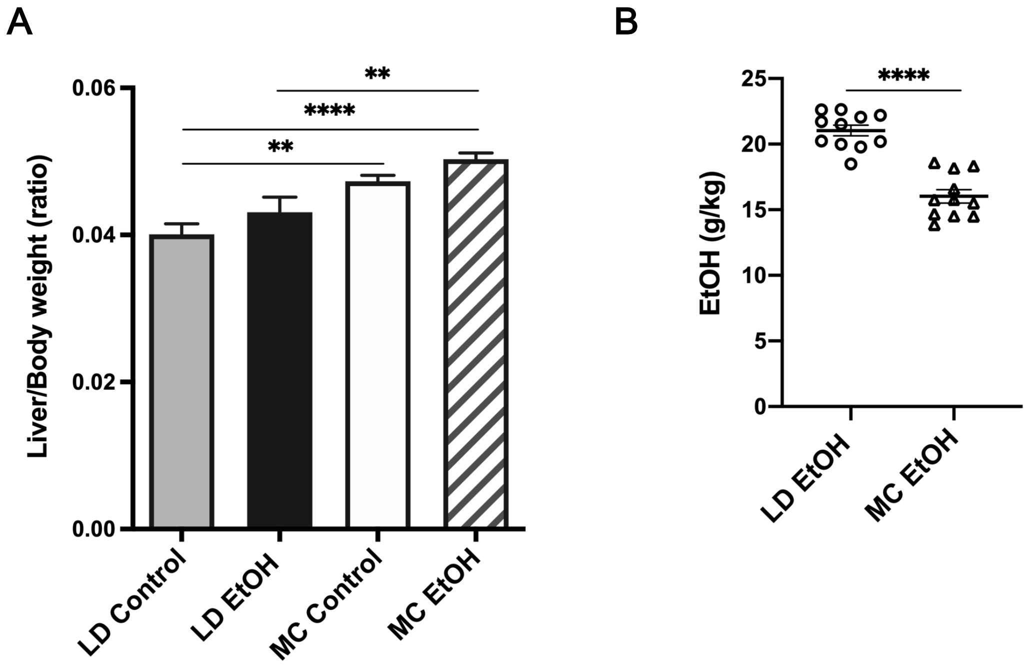

(A) Mice were subject to LD control (LD Control), LD ethanol (LD EtOH), regular chow diet (MC Control) and chronic ethanol exposure as per MC model (MC EtOH). The data represent the mean ± SEM, ***p* < 0.01; *****p* < 0.0001 (2-way ANOVA with Tukey's test, n = 10 mice LD group, n = 52 mice MC group).

(B) Mice were subject to LD control (LD Control), LD ethanol (LD EtOH), regular chow diet (MC Control) and chronic ethanol exposure as per MC model (MC EtOH). LD diet and MC EtOH (ethanol in drinking water) were freshly prepared every day. The body weights of all mice and the amounts of ethanol consumption were measured every day. The data represent the mean ± SEM, *****p* < 0.0001 (*t*-test with the Holm-Sidak method, n = 5 mice per group).

Supplementary Figure 2

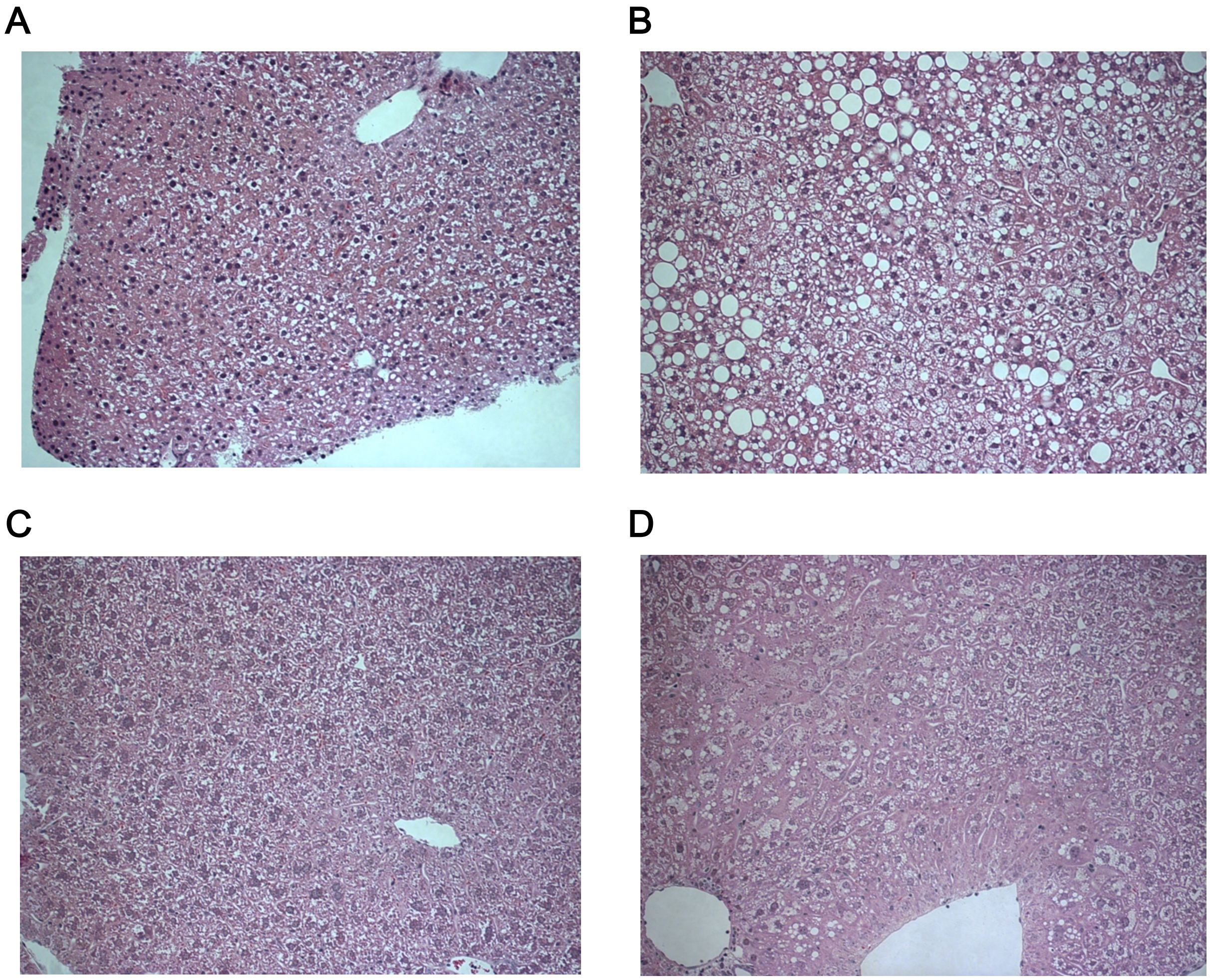

Liver tissue from mice subjected to LD control (Panel A), LD ethanol (Panel B), regular chow diet (Panel C) and chronic ethanol exposure as per MC model (Panel D) was fixed in 10% formalin for 24 hours, embedded in paraffin and stained with standard Hematoxylin and Eosin (H&E). Representative pictures x2000 were taken.

Supplementary Figure 3

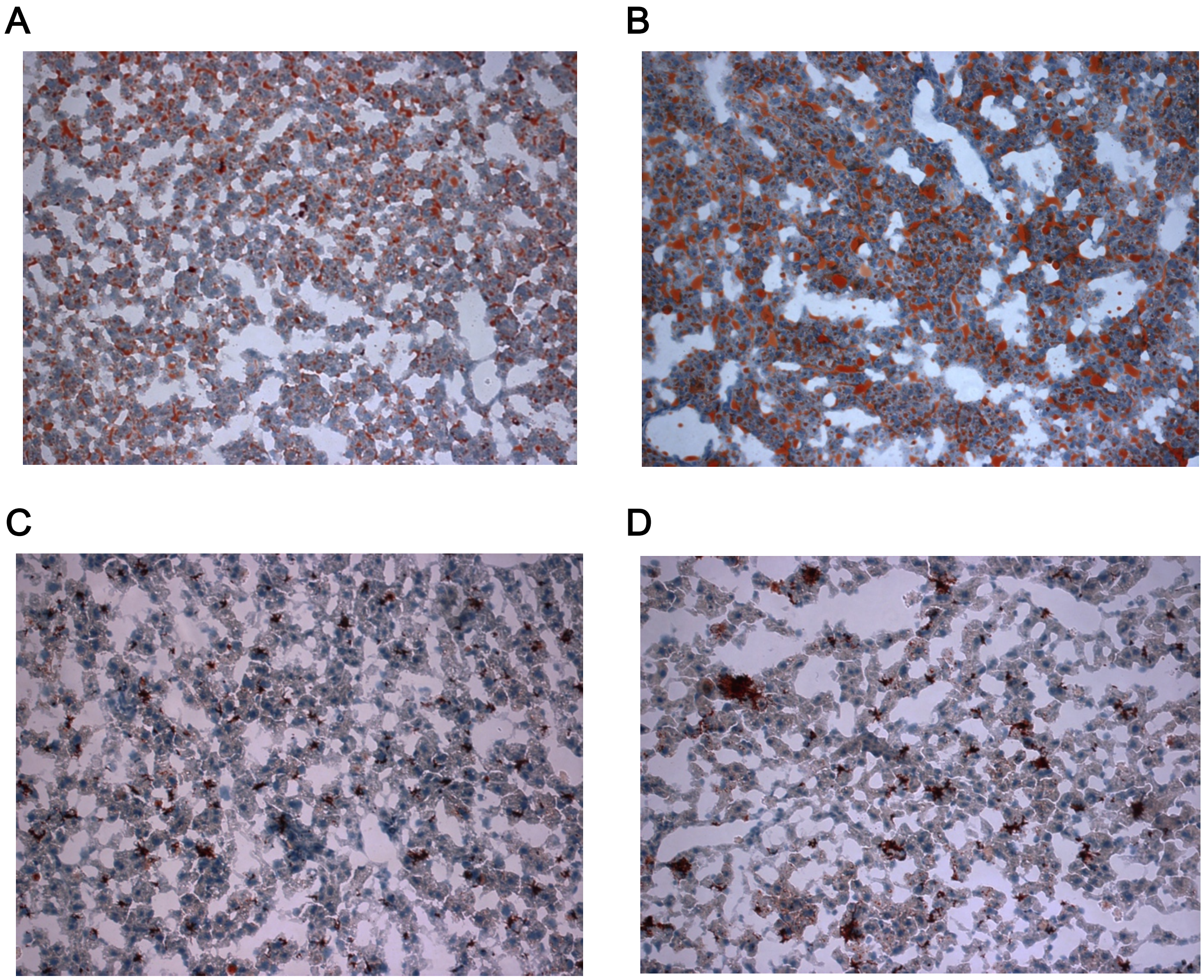

Liver tissue from mice subjected to LD control (Panel A), LD ethanol (Panel B), regular chow diet (Panel C) and chronic ethanol exposure as per MC model (Panel D) I were fixed in Tissue-Tek Optimal Cutting Temperature (OCT) compound (Sakura Inc, CA), processed and stained with Oil Red O. Representative pictures x2000 were taken.

Supplementary Table 1. List of DEGs (differential expressed genes) induced by alcohol and diet type

MC EtOH vs. MC Control		MC Control vs. LD Control		LD EtOH vs. LD Control		MC EtOH vs. LD EtOH	
Rgs16	regulator of G-protein signaling 16	Fabp5l2	fatty acid binding protein 5-like 2	Arhgef16	Rho guanine nucleotide exchange factor (GEF) 16	Lrp2bp	Lrp2 binding protein
Mogat1	monoacylglycerol O-acyltransferase 1	Adamdec1	ADAM-like, decysin 1	Scd3	stearoyl-coenzyme A desaturase 3	Dact1	disheveled-binding antagonist of beta-catenin 1
Zfp872	zinc finger protein 872	Ncam2	neural cell adhesion molecule 2	Hmgcr	3-hydroxy-3-methylglutaryl-Coenzyme A reductase	Sult3a1	sulfotransferase family 3A, member 1
Lurap1	leucine rich adaptor protein 1	Mirg	miRNA containing gene	Fads2	fatty acid desaturase 2	Sfrp1	secreted frizzled-related protein 1
				Noct	nocturnin (Ccr4)	Grcc10	gene rich cluster, C10 gene
Sult1e1	sulfotransferase family 1E, member 1	Afp	alpha fetoprotein	Pklr	pyruvate kinase liver and red blood cell	Cd207	CD207 antigen
Chil3	chitinase-like 3	Krt23	keratin 23	Gstp3	glutathione S-transferase pi 3	Cyp2c54	cytochrome P450, family 2, subfamily c, polypeptide 54
A1bg	alpha-1-B glycoprotein	Marco	macrophage receptor with collagenous structure	Hsbp1l1	heat shock factor binding protein 1-like 1	Sult2a7	sulfotransferase family 2A, member 7
		Slc39a5	solute carrier family 39 (metal ion transporter), member 5	Gstm3	glutathione S-transferase, mu 3	Slco1a1	solute carrier organic anion transporter family, member 1a1
		Cyp4a14	cytochrome P450, family 4, subfamily a, polypeptide 14	Prss8	protease, serine 8		
		Elovl3	elongation of very long chain fatty acids-like 3	Aqp4	aquaporin 4	Sprr1a	small proline-rich protein 1A
		Cyp4a31	cytochrome P450, family 4, subfamily a, polypeptide 31	Cyp17a1	cytochrome P450, family 17, subfamily a, polypeptide 1	Nr4a1	nuclear receptor subfamily 4, group A, member 1
				Scd1	stearoyl-Coenzyme A desaturase 1	Ppp1r3g	protein phosphatase 1, regulatory subunit 3G
				Fasn	fatty acid synthase	Aqp4	aquaporin 4
				Atp2b2	ATPase, Ca++ transporting, plasma membrane 2	Dbp	D site albumin promoter binding protein
				Slc22a29	solute carrier family 22. member 29	Gdf15	growth differentiation factor 15
				Apoa4	apolipoprotein A-IV	Sult2a3	sulfotransferase family 2A, member 3
				Slc2a4	solute carrier family 2 (facilitated glucose transporter), member 4	Fgf21	fibroblast growth factor 21

Supplementary Table 1. Continued

MC EtOH vs. MC Control		MC Control vs. LD Control		LD EtOH vs. LD Control		MC EtOH vs. LD EtOH	
				Nr4a1	nuclear receptor subfamily 4, group A, member 1	Nr1d1	nuclear receptor subfamily 1, group D, member 1
				Ppp1r3g	protein phosphatase 1, regulatory subunit 3G	Cyp17a1	cytochrome P450, family 17, subfamily a, polypeptide 1
				Fgf21	fibroblast growth factor 21	Zfp36	zinc finger protein 36
				Pdgfrl	platelet-derived growth factor receptor-like		
				Sprr1a	small proline-rich protein 1A		
				Slco1a1	solute carrier organic anion transporter family, member 1a1		
				Ikzf3	IKAROS family zinc finger 3		
				Cyp2c54	cytochrome P450, family 2, subfamily c, polypeptide 54		
				Clec2h	C-type lectin domain family 2, member h		
				Sult2a7	sulfotransferase family 2A, member 7		
				Sult3a1	sulfotransferase family 3A, member 1		
				Prok1	prokineticin 1		

* Red font, up; Blue font, down; Green font, up or down from different comparisons

Supplementary Table 2. List of DEGs analyzed by the four-way comparison

LD EtOH vs. LC and MC vs. LC		MC vs. LC and MC EtOH vs. LD EtOH		MC EtOH vs. MC and LD EtOH vs. LC		MC EtOH vs. MC and LD EtOH vs. LC and MC vs. LC		MC EtOH vs. MC and MC EtOH vs. LD EtOH	
Slc34a2	solute carrier family 34 (sodium phosphate), member 2	Srgap3	SLIT-ROBO Rho GTPase activating protein 3	**Elovl6**	elongation of very long chain fatty acids-like 6	**Pnpla3**	patatin-like phospholipase domain containing 3	**Lcn2**	lipocalin 2
Cyp2b10	cytochrome P450, family 2, subfamily b, polypeptide 10	Sult5a1	sulfotransferase family 5A, member 1	**Sult2a3**	sulfotransferase family2A, member 3				
Aacs	acetoacetyl-CoA synthetase	Sult3a2	sulfotransferase family 3A, member 2	**Mup11**	Major urinary protein 11				
Ugt1a5	UDP glucuronosyltransferase 1 family, polypeptide A5	A1bg	alpha-1-B glycoprotein						
Cyp3a44	cytochrome P450, family 3, subfamily a, polypeptide 44								
Dntt	deoxynucleotidyltransferase, terminal								
Cyp26a1	cytochrome P450, family 26, subfamily a, polypeptide 1								
Prom1	prominin 1								
Chrna4	cholinergic receptor, nicotinic, alpha polypeptide 4								
Cyp3a41a	cytochrome P450, family 3, subfamily a, polypeptide 41A								
Slc6a16	solute carrier family 6, member 16								

* Red font, up; Blue font, down; Green font, up or down from different comparisons

Supplementary Table 3. Fluorochrome conjugated antibodies used for Flow cytometry analysis

Abs	Clone	Fluorochromes	Dilution
**CD45**	30-F11	APC-Cy7	1:200
**CD3**	145-2C11	PE-CF594	1:200
**CD19**	1D3	PE-CF594	1:200
**NK1.1**	PK136	PE-CF594	1:200
**Ly6G**	1A8	AF 700	1:200
**PDCA1**	927	AF 488	1:200
**MHCII**	M5/114.15.2	PE-Cy7	1:400
**CD11c**	N418	BV 650	1:200
**CD11b**	M1/70	BV 421	1:200
**F4/80**	BM8	APC	1:100

Supplementary Table 4. List of TaqMan primers used for RT-qPCR

Name (Mouse)	Gene abbreviation	Gene Name	TaqMan ID	Conventional Forward	Conventional Reverse
B2M	B2m	beta-2 microglobulin	Mm00437762_m1	TTCTGGTGCTTGTCTCACTGA	CAGTATGTTCGGCTTCCCATTC
ACTB	Actb	actin, beta	Mm02619580_g1	ACCCGCCTCACATTGAAATCC	GGCGTATGTATCAGTCTCAGTG
ASMA	Acta2	actin, alpha 2, smooth muscle, aorta	Mm00725412_s1	GGCACCACTGAACCCTAAGG	ACAATACCAGTTGTACGTCCAGA
COL1A1	Col1a1	collagen, type I, alpha 1	Mm00801666_g1	CTGGCGGTTCAGGTCCAAT	TTCCAGGCAATCCACGAGC
COL1A2	Col1a2	collagen, type I, alpha 2	Mm00483888_m1	AAGGGTGCTACTGGACTCCC	TTGTTACCGGATTCTCCTTTGG
PDGFRb	Pdgfrb	platelet-derived growth factor receptor, beta polypeptide	Mm00435546_m1	AGGAGTGATACCAGCTTTAGTCC	CCGAGCAGGTCAGAACAAAGG
TGFb1	Tgfb1	transforming growth factor, beta 1	Mm01178820_m1	CTCCCGTGGCTTCTAGTGC	GCCTTAGTTTGGACAGGATCTG
IL-1α	Il1a	interleukin 1, alpha	Mm00439620_m1	CGAAGACTACAGTTCTGCCATT	GACGTTTCAGAGGTTCTCAGAG
IL-1β	Il1b	interleukin 1, beta	Mm00434228_m1	GAAATGCCACCTTTTGACAGTG	TGGATGCTCTCATCAGGACAG
IL-6	Il6	interleukin 6	Mm00446190_m1	CTGCAAGAGACTTCCATCCAG	AGTGGTATAGACAGGTCTGTTGG
IL-7	Il7	interleukin 7	Mm01295803_m1	TTCCTCCACTGATCCTTGTTCT	AGCAGCTTCCTTTGTATCATCAC
TNFα	Tnf	tumor necrosis factor alpha	Mm00443258_m1	CCTGTAGCCCACGTCGTAG	GGGAGTAGACAAGGTACAACCC
ICAM-1	Icam1	intercellular adhesion molecule 1	Mm00516023_m1	TGCCTCTGAAGCTCGGATATAC	TCTGTCGAACTCCTCAGTCAC
ICAM-2	Icam2	intercellular adhesion molecule 2	Mm00494862_m1	GCAGGACAACCAAATGGTCAT	AGAACAGCAGTATTGACACCAC
VCAM-1	Vcam1	vascular cell adhesion molecule 1	Mm01320970_m1	TTCGGTTGTTCTGACGTGTG	TACCACCCCATTGAGGGGAC
E-selectin	Sele	selectin, endothelial cell	Mm00441278_m1	ATGAAGCCAGTGCATACTGTC	CGGTGAATGTTTCAGATTGGAGT
P-selectin	Selplg	selectin, platelet ligand	Mm01204601_m1	CCACCGAAGTCCCTTCCAC	GCCGCTGTCAGGTAAGTGA
JAM-A	F11r	F11 receptor	Mm00554113_m1	TCTCTTCACGTCTATGATCCTGG	TTTGATGGACTCGTTCTCGGG
JAM-B	Jam2	junction adhesion molecule 2	Mm00470197_m1	GTGCCCACTTCTGTTATGACTG	TTCCCTAGCAAACTTGTGCCA
JAM-C	Jam3	junction adhesion molecule 3	Mm00499214_m1	CTTGCATCATTACGGACTCACA	GCCAGGTCTCCTTGAATCTTGTT
CCL2	Ccl2	chemokine (C-C motif) ligand 2	Mm00441242_m1	TAAAAACCTGGATCGGAACCAAA	GCATTAGCTTCAGATTTACGGGT

Supplementary Table 4. Continued

<table>
<tr><th>Name (Mouse)</th><th>Gene abbreviation</th><th>Gene Name</th><th>TaqMan ID</th><th>Conventional Forward</th><th>Conventional Reverse</th></tr>
<tr><td>CCL3</td><td>Ccl3</td><td>chemokine (C-C motif) ligand 3</td><td>Mm00441259_g1</td><td>TGTACCATGACACTCTGCAAC</td><td>CAACGATGAATTGGCGTGGAA</td></tr>
<tr><td>CCL4</td><td>Ccl4</td><td>chemokine (C-C motif) ligand 4</td><td>Mm00443111_m1</td><td>TTCCTGCTGTTTCTCTTACACCT</td><td>CTGTCTGCCTCTTTTGGTCAG</td></tr>
<tr><td>CCL5</td><td>Ccl5</td><td>chemokine (C-C motif) ligand 5</td><td>Mm01302427_m1</td><td>GCTGCTTTGCCTACCTCTCC</td><td>TCGAGTGACAAACACGACTGC</td></tr>
<tr><td>CCL7</td><td>Ccl7</td><td>chemokine (C-C motif) ligand 7</td><td>Mm00443113_m1</td><td>GCTGCTTTCAGCATCCAAGTG</td><td>CCAGGGACACCGACTACTG</td></tr>
<tr><td>CCL19</td><td>Ccl19</td><td>chemokine (C-C motif) ligand 19</td><td>Mm00839967_g1</td><td>GGGGTGCTAATGATGCGGAA</td><td>CCTTAGTGTGGTGAACACAACA</td></tr>
<tr><td>CCL20</td><td>Ccl20</td><td>chemokine (C-C motif) ligand 20</td><td>Mm01268754_m1</td><td>GCCTCTCGTACATACAGACGC</td><td>CCAGTTCTGCTTTGGATCAGC</td></tr>
<tr><td>CCL21A</td><td>Ccl21a</td><td rowspan="2">chemokine (C-C motif) ligand 21</td><td rowspan="2">Mm03646971_gH</td><td rowspan="2">GTGATGGAGGGGGTCAGGA</td><td rowspan="2">GGGATGGGACAGCCTAAACT</td></tr>
<tr><td>CCL21B</td><td>Ccl21b</td></tr>
<tr><td>CCL25</td><td>Ccl25</td><td>chemokine (C-C motif) ligand 25</td><td>Mm00436443_m1</td><td>GAGGGCGATGAGAATCTTGAC</td><td>TCCTCACGCTTGTACTGTTGG</td></tr>
<tr><td>CCL27</td><td>Ccl27</td><td>chemokine (C-C motif) ligand 27</td><td>Mm04206819_gH</td><td>AGGAGGATTGTCCACATGGAA</td><td>CTTGGCGTTCTAACCACCGA</td></tr>
<tr><td>CCL28</td><td>Ccl28</td><td>chemokine (C-C motif) ligand 28</td><td>Mm00445039_m1</td><td>AGAGTGAGTTCATGCAGCATC</td><td>CTGCTTCAAAGTACGATTGTGC</td></tr>
<tr><td>CXCL9</td><td>Cxcl9</td><td>chemokine (C-X-C motif) ligand 9</td><td>Mm00434946_m1</td><td>TCCTTTTGGGCATCATCTTCC</td><td>TTTGTAGTGGATCGTGCCTCG</td></tr>
<tr><td>CXCL10</td><td>Cxcl10</td><td>chemokine (C-X-C motif) ligand 10</td><td>Mm00445235_m1</td><td>CCAAGTGCTGCCGTCATTTTC</td><td>TCCCTATGGCCCTCATTCTCA</td></tr>
<tr><td>CXCL11</td><td>Cxcl11</td><td>chemokine (C-X-C motif) ligand 11</td><td>Mm00444662_m1</td><td>GGCTTCCTTATGTTCAAACAGGG</td><td>GCCGTTACTCGGGTAAATTACA</td></tr>
<tr><td>CXCL12</td><td>Cxcl12</td><td>chemokine (C-X-C motif) ligand 12</td><td>Mm00445553_m1</td><td>TGCATCAGTGACGGTAAACCA</td><td>CACAGTTTGGAGTGTTGAGGAT</td></tr>
</table>
